# Asymptotic Expansion and Weak Approximation for a Stochastic Control Problem on Path Space

**DOI:** 10.3390/e26020119

**Published:** 2024-01-29

**Authors:** Masaya Kannari, Riu Naito, Toshihiro Yamada

**Affiliations:** 1 Aflac Life Insurance Japan Ltd., Tokyo 163-0456, Japan; masaya.kannari@gmail.com; 2 Asset Management One, Co., Ltd., Tokyo 100-0005, Japan; riu.naito@gmail.com; 3 Graduate School of Economics, Hitotsubashi University, Tokyo 186-8601, Japan

**Keywords:** stochastic optimization, relative entropy, Monte Carlo simulation, asymptotic expansion, weak approximation

## Abstract

The paper provides a precise error estimate for an asymptotic expansion of a certain stochastic control problem related to relative entropy minimization. In particular, it is shown that the expansion error depends on the regularity of functionals on path space. An efficient numerical scheme based on a weak approximation with Monte Carlo simulation is employed to implement the asymptotic expansion in multidimensional settings. Throughout numerical experiments, it is confirmed that the approximation error of the proposed scheme is consistent with the theoretical rate of convergence.

## 1. Introduction

Constructing an efficient algorithm for the following stochastic control problem associated with a relative entropy minimization (1) is an interesting topic in various areas, such as probability theory, statistical physics, economics and financial mathematics:

(1) $$\left\{\begin{array}{c} \underset{h}{\inf}\left\{E\left[\frac{1}{2}\int_{0}^{T}{\left|h_{s}\right|}^{2}ds\right]+E\left[f\left(X_{T}^{x,\varepsilon ,\alpha h}\right)\right]\right\}\\ dX_{t}^{x,\varepsilon ,\alpha h}=b\left(X_{t}^{x,\varepsilon ,\alpha h}\right)dt+\varepsilon \sigma \left(X_{t}^{x,\varepsilon ,\alpha h}\right)\left[dW_{t}+\alpha h_{t}dt\right],\;\;X_{0}^{x,\varepsilon ,\alpha h}=x\in R^{N}. \end{array}\right.$$

Problem (1) appears in the risk-sensitive stochastic control problem, as studied in [1,2,3,4,5,6], where the optimal control is given by minimizing the cost depending on the risk-sensitivity of the policy maker. One of applications related to the problem (1) is the rare event simulation [7,8] in statistical physics, in which accurate approximations of rare event probabilities are studied. In the rare event simulation, importance sampling techniques are proposed by solving (1) through the variational representation based on the large deviation theory (see [9]). Moreover, the relation between the optimal control and data assimilation problems are discussed in [10].

In particular in finance, (1) is closely related to pricing and hedging problems in utility indifference pricing in incomplete market (see [11,12,13,14,15], for example). Since there is no closed-form solution for the stochastic control problem of utility indifference pricing in most cases, various numerical methods for computing indifference prices have been developed. For example, in [11], the mean-variance expansion for utility indifference pricing is proposed by using an expansion approach through Girsanov transformation. In [12], the author provides an alternative approach to the analysis of [11] by using the asymptotic expansion of the corresponding quadratic backward stochastic differential equation. The mean-variance expansion proposed in [11] is generalized in [14] to cover a multidimensional path-dependent payoff in Itô process markets. In [15], the authors extended the results of [11,14] for the case of non-smooth payoffs and apply pricing problems of power derivatives.

In the implementation of the mean-variance expansion of [11,14,15] numerically, a simple approach for computing the mean and the variance terms will be the use of the Euler–Maruyama discretization scheme for stochastic differential equations (SDEs). However, this requires many number of time steps to obtain an accurate result, since it is a first-order time discretization scheme. In other words, for a small number of time steps *n*, the error term by the Euler–Maruyama discretization may affect the total approximation error, including the mean-variance expansion error and the discretization error. Thus, it is important to improve the convergence rate of approximations for the mean and the variance terms in order to construct an efficient algorithm.

There have been extensive studies on asymptotic expansion methods for small noise diffusions with Malliavin calculus (for instance, [16,17,18]). Moreover, by extending these results, high-order discretization methods for SDEs are developed in various papers (for example, [19,20,21,22,23]). In particular, ref. [24] introduced a new high-order approximation method with respect to a small noise parameter ε and a number of discretization time steps *n* and implemented the method by deep learning.

In this paper, we show a precise error estimate of the mean-variance expansion of the stochastic control problem under various conditions on functionals on path space based on asymptotic expansion and Malliavin calculus. In particular, we prove the novel fact that the expansion error depends on the regularity of a target functional, which is an extended result of [11,14,15]. Then, we implement the mean-variance expansion by using the asymptotic expansion and weak approximation to achieve the high-order approximation error with respect to ε and *n* based on [24]. Numerical experiments confirm the theoretical convergence rate of the proposed method.

The organization of the paper is as follows. After introducing the notations and settings, we provide the main theorem and the approximation method in Section 2. Section 3 shows numerical examples of the proposed method. We conclude the paper in Section 4.

## 2. Asymptotic Expansion and Weak Approximation of Stochastic Control Problems

Let $C_{b}^{\infty }\left(R^{n};R^{m}\right)$ be the space of infinitely continuously differentiable functions $f:R^{n}\to R^{m}$ with bounded derivatives of all orders. We write $C_{b}^{\infty }\left(R^{n}\right)$ for $C_{b}^{\infty }\left(R^{n};R\right)$. Let $C_{\mathrm{Lip}}\left(R^{n}\right)$ be the space of Lipschitz continuous functions $f:R^{n}\to R$ with the Lipschitz constant $C_{\mathrm{Lip}}\left[f\right]$. Let $B_{b}\left(R^{n}\right)$ be the space of bounded Borel measurable functions $f:R^{n}\to R$. For $f\in B_{b}\left(R^{n}\right)$, we define ${∥f∥}_{\infty }:=\sup_{x\in R^{n}}\left|f\left(x\right)\right|$.

Let $\Omega =C_{0}\left(\left[0,T\right];R^{d}\right)=\left\{w:\left[0,T\right]\to R^{d};\;\mathrm{continuous},\;w\left(0\right)=0\right\}$, let B(Ω) be the Borel field over Ω, and let P be the Wiener measure P:B(Ω)→[0,1]. Let F be the completion of B(Ω) with respect to P. Let $W={\left\{W_{t}\right\}}_{0\leq t\leq T}$ be a *d*-dimensional Brownian motion on the probability space (Ω,F,P). Let ${\left\{F_{t}\right\}}_{0\leq t\leq T}$ be the filtration generated by *W*. We assume that ${\left\{F_{t}\right\}}_{0\leq t\leq T}$ contains the P-null sets of F. For a random variable $Y:\Omega \to R^{N}$ on the probability space (Ω,F,P), let E[Y] denote the expectation of *Y* and let Var[Y] denote the variance of *Y* and let ${∥X∥}_{p}:={E[|X|}^{p}{]}^{1/p}$, for p≥1. We define the space A as $A:=\{X:\Omega \times \left[0,T\right]\to R^{d};\;\left\{F_{t}\right\}-\mathrm{adapted},\;E[\int_{0}^{T}|X_{s}{|}^{2}ds]<\infty \}$.

We prepare notation from Malliavin calculus. Let $D^{\infty }\left(\Omega \right)$ denote the set of smooth Wiener functionals F:Ω→R in the sense of Malliavin. Let $F\in {\left(D^{\infty }\left(\Omega \right)\right)}^{N}$ be a nondegenerate Wiener functional. Then, for $G\in D^{\infty }\left(\Omega \right)$ and a multi-index $\alpha =\left(\alpha_{1},\ldots ,\alpha_{\ell }\right)\in {\left\{1,\ldots ,N\right\}}^{\ell }$, ℓ∈N, there exists $H_{\alpha }\left(F,G\right)\in D^{\infty }\left(\Omega \right)$ such that:

(2) $$\begin{array}{c} E\left[\partial^{\alpha }f\left(F\right)G\right]=E\left[f\left(F\right)H_{\alpha }\left(F,G\right)\right] \end{array}$$

for all $f\in C_{b}^{\infty }\left(R^{N}\right)$. For more details on Malliavin calculus, see [25,26].

We consider an *N*-dimensional diffusion driven by *W*: for 0≤t≤s≤T:

(3) $$\begin{array}{c} dX_{s}^{t,x,\varepsilon }=b\left(X_{s}^{t,x,\varepsilon }\right)ds+\varepsilon \sigma \left(X_{s}^{t,x,\varepsilon }\right)dW_{s},\;\;\;X_{t}^{t,x,\varepsilon }=x\in R^{N}, \end{array}$$

where $b,\sigma_{i}\in C_{b}^{\infty }\left(R^{N};R^{N}\right)$, i=1,…,d and ε∈(0,1]. We assume that $\sigma =[\sigma_{1},\ldots ,\sigma_{d}]$ satisfies the uniform elliptic condition. For notational simplicity, we write $X_{t}^{x,\varepsilon }$ for $X_{t}^{0,x,\varepsilon }$, 0≤t≤T, $x\in R^{N}$.

Let γ>0. It is known that the free energy of the small noise diffusion has the variational representation:

(4) $$\begin{array}{c} -\frac{1}{\gamma }\log E\left[\exp\left\{-\gamma f\left(X_{T}^{x,\varepsilon }\right)\right\}\right]=\underset{h\in A}{\inf}\left\{E\left[\frac{1}{2}\int_{0}^{T}{\left|h_{s}\right|}^{2}ds\right]+E\left[f\left(X_{T}^{x,\varepsilon ,\sqrt{\gamma }h}\right)\right]\right\} \end{array}$$

where $X^{x,\varepsilon ,\sqrt{\gamma }h}$ is a stochastic system with a control process h∈A:

(5) $$\begin{array}{c} dX_{t}^{x,\varepsilon ,\sqrt{\gamma }h}=b\left(X_{t}^{x,\varepsilon ,\sqrt{\gamma }h}\right)dt+\varepsilon \sigma \left(X_{t}^{x,\varepsilon ,\sqrt{\gamma }h}\right)\left[dW_{t}+\sqrt{\gamma }h_{t}dt\right],\;\;X_{0}^{x,\varepsilon ,\sqrt{\gamma }h}=x\in R^{N}. \end{array}$$

The main result is given as follows.

**Theorem** **1.** *It holds that:*

(6) $$\begin{array}{cc} \; & -\frac{1}{\gamma }\log E\left[\exp\left\{-\gamma f\left(X_{T}^{x,\varepsilon }\right)\right\}\right]=E\left[f\left(X_{T}^{x,\varepsilon }\right)\right]-\frac{\gamma }{2}\mathrm{Var}\left[f\left(X_{T}^{x,\varepsilon }\right)\right]+E_{\gamma ,\varepsilon }, \end{array}$$

*where:*

(7) $$E_{\gamma ,\varepsilon }=\left\{\begin{array}{cc} O(\gamma^{2}) & \mathrm{if}\;\;f\in B_{b}\left(R^{N}\right),\;\\ O(\gamma^{2}\varepsilon^{3}) & \mathrm{if}\;\;f\in C_{\mathrm{Lip}}\left(R^{N}\right)\cup C_{b}^{1}\left(R^{N}\right),\;\\ O(\gamma^{2}\varepsilon^{4}) & \mathrm{if}\;\;f\in \cup_{k\geq 2}C_{b}^{k}\left(R^{N}\right). \end{array}\right.$$

**Remark** **1.** *Theorem 1 provides a sharp asymptotic expansion for the solution of the stochastic control problem for the small noise diffusion, while the direct estimate of the left-hand side of (6) can cause inefficient computation, which is reported in [7,8]. In particular, Theorem 1 provides the theoretical approximation order with respect to both γ and ε for each class of test functions f, which cannot be obtained from the asymptotic analysis in the context of the risk-sensitive control problems in [1,2,3] and the indifference pricing problems in [11,14,15]. In the proof of Theorem 1 below, we will take another approach to show the sharp asymptotic expansion bounds (7), and Malliavin calculus plays a crucial role in the error estimate.*

**Remark** **2.** *In the utility indifference pricing problems, γ is regarded as the risk-aversion parameter of an investor’s exponential utility function $U\left(x\right)=-e^{-\gamma x}$ and is typically assumed to be small as γ≈0 (see [11,14,15] for more details), which is a natural setting that the investor is not far from risk-neutral (γ=0 corresponds to the case that the investor is risk-neutral). Thus, the mean-variance expansion is interpreted as the expansion around the sum of the risk-neutral price $E[f\left(X_{T}^{x,\varepsilon }\right)]$ and the risk-aversion discount effect $-\frac{\gamma }{2}\mathrm{Var}\left[f\left(X_{T}^{x,\varepsilon }\right)\right]$. Theorem 1 tells us that the expansion error depends not only on the risk-aversion parameter γ but also on the smoothness of the payoff function f and the small noise parameter ε, which is a significant information in computing indifference prices in practice.*

**Proof of Theorem** **1.** We introduce a perturbed process with δ>0:

(8) $$\begin{array}{c} dX_{t}^{x,\varepsilon ,\delta h}=b\left(X_{t}^{x,\varepsilon ,\delta h}\right)dt+\varepsilon \sigma \left(X_{t}^{x,\varepsilon ,\delta h}\right)\left[dW_{t}+\delta h_{t}dt\right],\;\;\;X_{0}^{x,\varepsilon ,\delta h}=x, \end{array}$$

in order to expand the minimization problem:

(9) $$\begin{array}{c} \underset{h\in A}{\inf}\left\{E\left[\frac{1}{2}\int_{0}^{T}{\left|h_{s}\right|}^{2}ds\right]+E\left[f\left(X_{T}^{x,\varepsilon ,\delta h}\right)\right]\right\}, \end{array}$$

which corresponds to (4) if we set $\delta =\sqrt{\gamma }$. For notational simplicity, hereafter, we assume N=d=1 without loss of generality.

The expansion of $X_{t}^{x,\varepsilon ,\delta h}$ in $D^{\infty }\left(\Omega \right)$ is calculated in the following way:

$$\begin{array}{cc} dX_{t}^{x,\varepsilon ,0} & =b\left(X_{t}^{x,\varepsilon ,0}\right)dt+\varepsilon \sigma \left(X_{t}^{x,\varepsilon ,0}\right)dW_{t}\left(=dX_{t}^{x,\varepsilon }\right),\\ d\frac{\partial }{\partial \delta }X_{t}^{x,\varepsilon ,\delta h} & =b^{\prime }\left(X_{t}^{x,\varepsilon ,\delta h}\right)\frac{\partial }{\partial \delta }X_{t}^{x,\varepsilon ,\delta h}dt+\varepsilon \sigma^{\prime }\left(X_{t}^{x,\varepsilon ,\delta h}\right)\frac{\partial }{\partial \delta }X_{t}^{x,\varepsilon ,\delta h}dW_{t}+\varepsilon \sigma \left(X_{t}^{x,\varepsilon ,\delta h}\right)h_{t}dt\\ \; & \;+\delta \varepsilon \sigma^{\prime }\left(X_{t}^{x,\varepsilon ,\delta h}\right)h_{t}\frac{\partial }{\partial \delta }X_{t}^{x,\varepsilon ,\delta h}dt, \end{array}$$

and so on. We introduce the Jacobian of $x\mapsto X^{x,\varepsilon ,\delta h}$, i.e., $Y_{t}^{x,\varepsilon ,\delta h}=\frac{\partial }{\partial x}X_{t}^{x,\varepsilon ,\delta h}$, whose dynamics are:

(10) $$\begin{array}{cc} \; & dY_{t}^{x,\varepsilon ,\delta h}=b^{\prime }\left(X_{t}^{x,\varepsilon ,\delta h}\right)Y_{t}^{x,\varepsilon ,\delta h}dt+\varepsilon \sigma^{\prime }\left(X_{t}^{x,\varepsilon ,\delta h}\right)Y_{t}^{x,\varepsilon ,\delta h}\left[dW_{t}+\delta h_{t}dt\right],\;\;Y_{0}^{x,\varepsilon ,\delta h}=1. \end{array}$$

We will use a notation $Y_{t}^{x,\varepsilon }=Y_{t}^{x,\varepsilon ,0}$.

The first-order term of the expansion of $E[f\left(X_{T}^{x,\varepsilon ,\delta h}\right)]$ with respect to δ is given by:

(11) $$\begin{array}{cc} E\left[{\left.f^{\prime }\left(X_{t}^{x,\varepsilon }\right)\frac{\partial }{\partial \delta }X_{t}^{x,\varepsilon ,\delta h}\right|}_{\delta =0}\right] & =E\left[f^{\prime }\left(X_{t}^{x,\varepsilon }\right)Y_{t}^{x,\varepsilon }\int_{0}^{t}{\left(Y_{s}^{x,\varepsilon }\right)}^{-1}\varepsilon \sigma \left(X_{s}^{x,\varepsilon }\right)h_{s}ds\right]\\ \; & =E\left[\int_{0}^{t}D_{s}f\left(X_{t}^{x,\varepsilon }\right)h_{s}ds\right]=E\left[f\left(X_{t}^{x,\varepsilon }\right)\int_{0}^{t}h_{s}dW_{s}\right], \end{array}$$

where $D_{t}F$, 0≤t≤T represents the Malliavin derivative process of $F\in D^{\infty }\left(\Omega \right)$ (for more details, please see [26]). Then, we have the following expansion:

(12) $$\begin{array}{c} E\left[f\left(X_{T}^{x,\varepsilon ,\delta h}\right)\right]=E\left[f\left(X_{T}^{x,\varepsilon }\right)\right]+\delta E\left[f\left(X_{T}^{x,\varepsilon }\right)\int_{0}^{T}h_{t}dW_{t}\right]+R_{x,\varepsilon ,\delta }^{h}\left(T\right), \end{array}$$

where $R_{x,\varepsilon ,\delta }^{h}\left(T\right)=\delta^{2}\int_{0}^{1}\left(1-\eta \right)\frac{\partial^{2}}{\partial \lambda^{2}}E\left[f\left(X_{T}^{x,\varepsilon ,\lambda h}\right)\right]{|}_{\lambda =\eta \delta }d\eta$, which satisfies that if $f\in B_{b}\left(R\right)$:

(13) $$\begin{array}{c} |R_{x,\varepsilon ,\delta }^{h}\left(T\right)|\leq \delta^{2}{∥f∥}_{\infty }C\left\{\frac{1}{\varepsilon^{2}}\underset{a\in (0,1]}{\sup}{∥{\left(\int_{0}^{T}Y_{T}^{x,\varepsilon ,ah}{\left(Y_{s}^{x,\varepsilon ,ah}\right)}^{-1}\varepsilon \sigma \left(X_{s}^{x,\varepsilon ,ah}\right)h_{s}ds\right)}^{2}∥}_{p}\right.\\ \left.\;\;\;+\frac{1}{\varepsilon }\underset{a\in (0,1]}{\sup}{∥\int_{0}^{T}Y_{T}^{x,\varepsilon ,ah}{\left(Y_{s}^{x,\varepsilon ,ah}\right)}^{-1}\varepsilon \sigma \left(X_{s}^{x,\varepsilon ,ah}\right)h_{s}\int_{0}^{s}Y_{s}^{x,\varepsilon ,ah}{\left(Y_{r}^{x,\varepsilon ,ah}\right)}^{-1}\varepsilon \sigma \left(X_{r}^{x,\varepsilon ,ah}\right)h_{r}drds∥}_{q}\right\}, \end{array}$$

or if $f\in C_{\mathrm{Lip}}\left(R\right)$:

(14) $$\begin{array}{c} |R_{x,\varepsilon ,\delta }^{h}\left(T\right)|\leq \delta^{2}{∥f^{\prime }∥}_{\infty }C\left\{\frac{1}{\varepsilon }\underset{a\in (0,1]}{\sup}\int_{0}^{T}Y_{T}^{x,\varepsilon ,ah}{\left(Y_{s}^{x,\varepsilon ,ah}\right)}^{-1}\varepsilon \sigma \left(X_{s}^{x,\varepsilon ,ah}\right)h_{s}ds{)}^{2}{∥}_{p}\right.\\ \left.\;\;\;+\underset{a\in (0,1]}{\sup}{∥\int_{0}^{T}Y_{T}^{x,\varepsilon ,ah}{\left(Y_{s}^{x,\varepsilon ,ah}\right)}^{-1}\varepsilon \sigma \left(X_{s}^{x,\varepsilon ,ah}\right)h_{s}\int_{0}^{s}Y_{s}^{x,\varepsilon ,ah}{\left(Y_{r}^{x,\varepsilon ,ah}\right)}^{-1}\varepsilon \sigma \left(X_{r}^{x,\varepsilon ,ah}\right)h_{r}drds∥}_{q}\right\}, \end{array}$$

or if $f\in \cup_{k\geq 2}C_{b}^{k}\left(R\right)$:

(15) $$\begin{array}{c} |R_{x,\varepsilon ,\delta }^{h}\left(T\right)|\leq \delta^{2}C\left\{∥f^{\prime \prime }{∥}_{\infty }\underset{a\in (0,1]}{\sup}{∥{\left(\int_{0}^{T}Y_{T}^{x,\varepsilon ,ah}{\left(Y_{s}^{x,\varepsilon ,ah}\right)}^{-1}\varepsilon \sigma \left(X_{s}^{x,\varepsilon ,ah}\right)h_{s}ds\right)}^{2}∥}_{p}\right.\\ \left.\;\;\;+∥f^{\prime }{∥}_{\infty }\underset{a\in (0,1]}{\sup}{∥\int_{0}^{T}Y_{T}^{x,\varepsilon ,ah}{\left(Y_{s}^{x,\varepsilon ,ah}\right)}^{-1}\varepsilon \sigma \left(X_{s}^{x,\varepsilon ,ah}\right)h_{s}\int_{0}^{s}Y_{s}^{x,\varepsilon ,ah}{\left(Y_{r}^{x,\varepsilon ,ah}\right)}^{-1}\varepsilon \sigma \left(X_{r}^{x,\varepsilon ,ah}\right)h_{r}drds∥}_{q}\right\}, \end{array}$$

for some C>0, p,q≥1 independent of *f*, ε and δ, through the Malliavin integration by parts formula of (2). By the Itô formula, it holds that:

(16) $$\begin{array}{c} f\left(X_{T}^{x,\varepsilon }\right)=E\left[f\left(X_{T}^{x,\varepsilon }\right)\right]+\int_{0}^{T}\left(\nabla P_{T-s}^{\varepsilon }f\right)\left(X_{s}^{x,\varepsilon }\right)\sigma^{\varepsilon }\left(X_{s}^{x,\varepsilon }\right)dW_{s}, \end{array}$$

where $P_{t}^{\varepsilon }f\left(\cdot \right)=E\left[f\left(X_{t}^{\cdot ,\varepsilon }\right)\right]$ and $\sigma^{\varepsilon }\left(\cdot \right)=\varepsilon \sigma \left(\cdot \right)$, which corresponds to the Clark–Ocone formula (see [26] for more detail). We should note that if *f* is a sufficiently smooth function, $\left(\nabla P_{T-s}^{\varepsilon }f\right)\left(x\right)\sigma^{\varepsilon }\left(x\right)$ is represented by:

(17) $$\begin{array}{cc} \; & \nabla P_{T-s}^{\varepsilon }f\left(x\right)\sigma^{\varepsilon }\left(x\right)=E\left[f^{\prime }\left(X_{T-s}^{x,\varepsilon }\right)Y_{T-s}^{x,\varepsilon }\right]\sigma^{\varepsilon }\left(x\right)\\ \; & =\frac{1}{T-s}E\left[\int_{0}^{T-s}f^{\prime }\left(X_{T-s}^{x,\varepsilon }\right)Y_{T-s}^{x,\varepsilon }{\left(Y_{r}^{x,\varepsilon }\right)}^{-1}\sigma^{\varepsilon }\left(X_{r}^{x,\varepsilon }\right)\sigma^{\varepsilon }{\left(X_{r}^{x,\varepsilon }\right)}^{-1}Y_{r}^{x,\varepsilon }dr\right]\sigma^{\varepsilon }\left(x\right)\\ \; & =\frac{1}{T-s}E\left[\int_{0}^{T-s}D_{r}f\left(X_{T-s}^{x,\varepsilon }\right)\sigma^{\varepsilon }{\left(X_{r}^{x,\varepsilon }\right)}^{-1}Y_{r}^{x,\varepsilon }dr\right]\sigma^{\varepsilon }\left(x\right) \end{array}$$

(18) $$\begin{array}{cc} \; & =\frac{1}{T-s}E\left[f\left(X_{T-s}^{x,\varepsilon }\right)\int_{0}^{T-s}\sigma^{\varepsilon }{\left(X_{r}^{x,\varepsilon }\right)}^{-1}Y_{r}^{x,\varepsilon }dW_{r}\right]\sigma^{\varepsilon }\left(x\right). \end{array}$$

Remark that in the case N≠d, we have a similar but a more general representation of (18) under the uniform ellipticity.

If we take a sequence of functions which approximates a Schwartz distribution which is regarded as a bounded measurable function, we have that there exists C>0 such that:

(19) $$\begin{array}{cc} \; & |\nabla P_{T-s}^{\varepsilon }f\left(x\right)\sigma^{\varepsilon }\left(x\right)|\leq \frac{C}{\sqrt{T-s}}{∥f∥}_{\infty },\;\;s\in \left[0,T\right],\;\;f\in B_{b}\left(R\right), \end{array}$$

and if we take a sequence of functions which approximates $f\in C_{\mathrm{Lip}}\left(R\right)$, by (17), there exists C>0 such that:

(20) $$\begin{array}{c} |\nabla P_{T-s}^{\varepsilon }f\left(x\right)\sigma^{\varepsilon }\left(x\right)|\leq C\varepsilon C_{\mathrm{Lip}}\left[f\right],\;\;s\in \left[0,T\right],\;\;f\in C_{\mathrm{Lip}}\left(R\right). \end{array}$$

Furthermore, it is obvious to apply (17) for the case *f* is smooth to obtain the desired estimate. To summarize the above discussion, we have the following gradient estimate for the diffusion semigroup which depends on the smoothness condition on *f*: there exists C>0 such that:

(21) $$|\left(\nabla P_{T-s}^{\varepsilon }f\right)\left(x\right)\sigma^{\varepsilon }\left(x\right)|\leq \left\{\begin{array}{cc} \frac{C}{\sqrt{T-s}}{∥f∥}_{\infty } & \mathrm{if}\;\;f\in B_{b}\left(R\right),\;\\ C\varepsilon C_{\mathrm{Lip}}\left[f\right] & \mathrm{if}\;\;f\in C_{\mathrm{Lip}}\left(R\right)\cup C_{b}^{1}\left(R\right),\;\\ C\varepsilon ∥f^{\prime }{∥}_{\infty } & \mathrm{if}\;\;f\in \cup_{k\geq 2}C_{b}^{k}\left(R\right), \end{array}\right.$$

for all s∈[0,T]. Therefore, we have:

(22) $$\begin{array}{c} E\left[f\left(X_{T}^{x,\varepsilon }\right)\int_{0}^{T}h_{s}dW_{s}\right]=E\left[\int_{0}^{T}\left(\nabla P_{T-s}^{\varepsilon }f\right)\left(X_{s}^{x,\varepsilon }\right)\sigma^{\varepsilon }\left(X_{s}^{x,\varepsilon }\right)h_{s}ds\right] \end{array}$$

and

$$\begin{array}{ccc} & & E\left[\frac{1}{2}\int_{0}^{T}{\left|h_{s}\right|}^{2}ds\right]+E\left[f\left(X_{T}^{x,\varepsilon ,\delta h}\right)\right]\\ & = & E\left[f\left(X_{T}^{x,\varepsilon }\right)\right]+\delta E\left[\int_{0}^{T}\left(\nabla P_{T-s}^{\varepsilon }f\right)\left(X_{s}^{x,\varepsilon }\right)\sigma^{\varepsilon }\left(X_{s}^{x,\varepsilon }\right)h_{s}ds\right]+E\left[\frac{1}{2}\int_{0}^{T}{\left|h_{s}\right|}^{2}ds\right]+R_{x,\varepsilon ,\delta }^{h}\left(T\right). \end{array}$$

By taking *h* as $h_{s}=-\delta \left(\nabla P_{T-s}^{\varepsilon }f\right)\left(X_{s}^{x,\varepsilon }\right)\sigma^{\varepsilon }\left(X_{s}^{x,\varepsilon }\right)$, and combining with (13)–(16) and (21), we have:

(23) $$\begin{array}{c} \underset{h\in A}{\inf}\left\{E\left[\frac{1}{2}\int_{0}^{T}{\left|h_{s}\right|}^{2}ds\right]+E\left[f\left(X_{T}^{x,\varepsilon ,\delta h}\right)\right]\right\}=E\left[f\left(X_{T}^{x,\varepsilon }\right)\right]-\frac{\delta^{2}}{2}\mathrm{Var}\left[f\left(X_{T}^{x,\varepsilon }\right)\right]+E_{\delta^{2},\varepsilon } \end{array}$$

with the error:

(24) $$E_{\delta^{2},\varepsilon }=\left\{\begin{array}{cc} O(\delta^{4}) & \mathrm{if}\;\;f\in B_{b}\left(R\right),\;\\ O(\delta^{4}\varepsilon^{3}) & \mathrm{if}\;\;f\in C_{\mathrm{Lip}}\left(R\right)\cup C_{b}^{1}\left(R\right),\;\\ O(\delta^{4}\varepsilon^{4}) & \mathrm{if}\;\;f\in \cup_{k\geq 2}C_{b}^{k}\left(R\right). \end{array}\right.$$

Finally, setting $\delta =\sqrt{\gamma }$, we have:

(25) $$\begin{array}{c} -\frac{1}{\gamma }\log E\left[\exp\left\{-\gamma f\left(X_{T}^{x,\varepsilon }\right)\right\}\right]=E\left[f\left(X_{T}^{x,\varepsilon }\right)\right]-\frac{\gamma }{2}\mathrm{Var}\left[f\left(X_{T}^{x,\varepsilon }\right)\right]+E_{\gamma ,\varepsilon }. \end{array}$$

□

**Remark** **3.** *We comment on the advantages of the Malliavin calculus approach (the asymptotic expansion approach [17,18,19] based on the Watanabe theory [16]) taken in the current paper. While (11) can be obtained from both the Girsanov transform approach [11] and the Malliavin calculus approach, the error estimate (24) and the result of Theorem 1 come from only the latter approach. Although the Girsanov transform approach is useful to derive the approximation itself, it only shows the error bound of the mean-variance expansion with respect to γ as in [11]. On the other hand, the Malliavin calculus approach provides a sharp error bound not only with respect to γ but also ε depending on the smoothness of the test function f. Hence, throughout the proof in Theorem 1, we adopted the unified derivation for the approximation term (11) and the residual term(s) (13)–(15) through Malliavin calculus. Moreover, the Malliavin calculus approach will be a powerful tool to approximate the mean and variance terms of the expansion in Theorem 1. We will see the usefulness of the approach in the following.*

In order to implement the asymptotic expansion of Theorem 1 numerically in multidimensional settings, we efficiently approximate the mean and the variance terms by a weak approximation method for the SDE (3).

We expand the *N*-dimensional diffusion process *X* as follows: for 0≤t≤s≤T:

(26) $$\begin{array}{c} X_{s}^{t,x,\varepsilon }=X_{s}^{t,x,0}+\varepsilon \frac{\partial }{\partial \varepsilon }X_{s}^{t,x,\varepsilon }{|}_{\varepsilon =0}+\varepsilon^{2}\frac{1}{2!}\frac{\partial^{2}}{\partial \varepsilon^{2}}X_{s}^{t,x,\varepsilon }{|}_{\varepsilon =0}+\cdots +\varepsilon^{k}\frac{1}{k!}\frac{\partial^{k}}{\partial \varepsilon^{k}}X_{s}^{t,x,\varepsilon }{|}_{\varepsilon =0}+\cdots \;\;\mathrm{in}\;{\left(D^{\infty }\left(\Omega \right)\right)}^{N}. \end{array}$$

Let $0=t_{0}<t_{1}<\cdots <t_{n}=T$, $t_{i+1}-t_{i}=T/n$, i=0,…,n−1. Here, we define ${\bar{X}}_{t_{i+1}}^{x,\varepsilon ,n}$ as:

(27) $$\begin{array}{cc} {\bar{X}}_{t_{i+1}}^{x,\varepsilon ,n} & ={\bar{X}}_{t_{i+1}}^{t_{i},{\bar{X}}_{t_{i}}^{t_{i},x,n},\varepsilon },\;\;{\bar{X}}_{t_{0}}^{x,\varepsilon ,n}=x\in R^{N}, \end{array}$$

where ${\bar{X}}_{s}^{t,x,\varepsilon }$, 0≤t≤s≤T is given by:

(28) $$\begin{array}{cc} {\bar{X}}_{s}^{t,x,\varepsilon } & =X_{s}^{t,x,0}+\varepsilon \frac{\partial }{\partial \varepsilon }X_{s}^{t,x,\varepsilon }{|}_{\varepsilon =0}. \end{array}$$

Moreover, we introduce the weight $W_{T}^{\varepsilon ,n}$ as:

(29) $$\begin{array}{c} W_{T}^{\varepsilon ,n}=\prod_{i=0}^{n-1}\vartheta_{t_{i},t_{i+1}}^{{\bar{X}}_{t_{i}}^{x,\varepsilon ,n},\varepsilon } \end{array}$$

where $\vartheta_{t,s}^{x,\varepsilon }$ satisfies that there exists C>0 such that:

(30) $$\begin{array}{c} |E\left[\varphi \left(X_{s}^{t,x,\varepsilon }\right)\right]-E\left[\varphi \left({\bar{X}}_{s}^{t,x,\varepsilon }\right)\vartheta_{t,s}^{x,\varepsilon }\right]|\;\leq C\varepsilon^{6}{∥\varphi ∥}_{\infty }{\left(s-t\right)}^{3}+C\sum_{p=0}^{4}\varepsilon^{2+p}{∥\nabla^{p}\varphi ∥}_{\infty }{\left(s-t\right)}^{3}, \end{array}$$

for all $\varphi \in C_{b}^{4}\left(R^{N}\right)$, $x\in R^{N}$ and s>t≥0. For more details on the derivation and the explicit form of the weight $\vartheta_{t,s}^{x,\varepsilon }$, see [24].

Using the scheme ${\bar{X}}_{T}^{x,\varepsilon ,n}$ and the weight $W_{T}^{\varepsilon ,n}$, we have the following approximation whose property again depends on the regularity.

**Corollary** **1.** *It holds that:*

(31) $$\begin{array}{cc} \; & -\frac{1}{\gamma }\log E\left[\exp\left\{-\gamma f\left(X_{T}^{x,\varepsilon }\right)\right\}\right]\\ & =E\left[f\left({\bar{X}}_{T}^{x,\varepsilon ,n}\right)W_{T}^{\varepsilon ,n}\right]-\frac{\gamma }{2}\left\{E\left[f{\left({\bar{X}}_{T}^{x,\varepsilon ,n}\right)}^{2}W_{T}^{\varepsilon ,n}\right]-E{\left[f\left({\bar{X}}_{T}^{x,\varepsilon ,n}\right)W_{T}^{\varepsilon ,n}\right]}^{2}\right\}+E_{\gamma ,\varepsilon ,n}, \end{array}$$

*where:*

(32) $$E_{\gamma ,\varepsilon ,n}=\left\{\begin{array}{cc} O\left(\gamma^{2}+\frac{\varepsilon^{2}}{n^{2}}\right) & \mathrm{if}\;\;f\in B_{b}\left(R^{N}\right),\;\\ O\left(\gamma^{2}\varepsilon^{3}+\frac{\varepsilon^{3}}{n^{2}}\right) & \mathrm{if}\;\;f\in C_{\mathrm{Lip}}\left(R^{N}\right)\cup C_{b}^{1}\left(R^{N}\right),\;\\ O\left(\gamma^{2}\varepsilon^{4}+\frac{\varepsilon^{3}}{n^{2}}\right) & \mathrm{if}\;\;f\in \cup_{k\geq 2}C_{b}^{k}\left(R^{N}\right). \end{array}\right.$$

**Proof of Corollary** **1.** By [24], each expectation is discretized with the order $O(1/n^{2})$ as follows:

(33) $$\begin{array}{c} \;\;\;E\left[f\left(X_{T}^{x,\varepsilon }\right)\right]-\frac{\gamma }{2}\left\{E\left[f{\left(X_{T}^{x,\varepsilon }\right)}^{2}\right]-E{\left[f\left(X_{T}^{x,\varepsilon }\right)\right]}^{2}\right\}\\ =E\left[f\left({\bar{X}}_{T}^{x,\varepsilon ,n}\right)W_{T}^{\varepsilon ,n}\right]-\frac{1}{2}\left\{E\left[f{\left({\bar{X}}_{T}^{x,\varepsilon ,n}\right)}^{2}W_{T}^{\varepsilon ,n}\right]-E{\left[f\left({\bar{X}}_{T}^{x,\varepsilon ,n}\right)W_{T}^{\varepsilon ,n}\right]}^{2}\right\}+{\bar{E}}_{\varepsilon ,n}, \end{array}$$

with:

(34) $${\bar{E}}_{\varepsilon ,n}=\left\{\begin{array}{cc} O\left(\frac{\varepsilon^{2}}{n^{2}}\right) & \mathrm{if}\;\;f\in B_{b}\left(R^{N}\right),\;\\ O\left(\frac{\varepsilon^{3}}{n^{2}}\right) & \mathrm{if}\;\;f\in C_{\mathrm{Lip}}\left(R^{N}\right)\cup C_{b}^{1}\left(R^{N}\right),\;\\ O\left(\frac{\varepsilon^{3}}{n^{2}}\right) & \mathrm{if}\;\;f\in \cup_{k\geq 2}C_{b}^{k}\left(R^{N}\right), \end{array}\right.$$

through the applicability of the Malliavin integration by parts in the global error analysis of the weak approximation analysis according to the regularity of *f*. Then, combining (33) with Theorem 1, the assertion is proved. □

**Remark** **4.** *As in the proof of Theorem 1, Malliavin integration by parts formula plays an important role to prove Corollary 1. The detail proof of the small noise expansion error is shown in [17,18] and the global error analysis of weak approximation error is provided in [19,20,21,22,23]. These results are essential to show the precise error estimate (34) depending on the regularity of the test function, which is an extension of the error estimate of [24].*

## 3. Numerical Examples

This section provides numerical experiments to show the validity of the proposed algorithm for indifference pricing problems.

Let (Ω,F,P) be a Wiener space (which is appropriately chosen in each subsection below) on which a Brownian motion is defined. We regard P as the physical probability measure. Let M be the set of equivalent martingale measures. Let U:R→R be the investor’s utility function given by U(x)=−exp(−γx), γ>0.

### 3.1. Indifference Pricing under Black–Scholes Model with a Lipschitz Payoff Function

We consider 2d-dimensional SDE (*d*-tradable assets $S^{s}=\left(S^{s,1},\ldots ,S^{s,d}\right)$ and *d*-nontradable assets $X^{x,\varepsilon }=\left(X^{x,1,\varepsilon },\ldots ,X^{x,d,\varepsilon }\right)$):

(35) $$\begin{array}{cc} dS_{t}^{s,i} & =\mu^{S}S_{t}^{s,i}dt+\sigma^{S}S_{t}^{s,i}dW_{t}^{2i-1},\;S_{0}^{s,i}=s_{i}\in R \end{array}$$

(36) $$\begin{array}{cc} dX_{t}^{x,i,\varepsilon } & =\mu^{X}X_{t}^{x,i,\varepsilon }dt+\varepsilon \sigma^{X}X_{t}^{x,i,\varepsilon }\left(\rho dW_{t}^{2i-1}+\sqrt{1-\rho^{2}}dW_{t}^{2i}\right),\;X_{0}^{x,i,\varepsilon }=x_{i}\in R, \end{array}$$

for i=1,…,d, $\mu^{S},\mu^{X},\rho \in R$ and $\sigma^{S},\sigma^{X}>0$, where $W=(W^{1},\ldots ,W^{2d})$ is a P-dimensional Brownian motion. The model of (35) and (36) referred to as the Black–Scholes model is widely used in financial institutions. We define Q∈M by:

(37) $$\begin{array}{c} \frac{dQ}{dP}=e^{\sum_{i=1}^{d}\left(mW_{T}^{2i-1}-\frac{1}{2}m^{2}T\right)+\sum_{i=1}^{d}\int_{0}^{T}\sqrt{\gamma }h_{t}^{i}dW_{t}^{2i}-\frac{1}{2}\int_{0}^{T}\gamma {\left|h_{t}\right|}^{2}dt}, \end{array}$$

for $m=\frac{r-\mu^{S}}{\sigma^{S}}$ and h∈A. Under a probability measure Q, we can rewrite the above SDE as:

(38) $$\begin{array}{cc} dS_{t}^{s,i} & =rS_{t}^{s,i}dt+\sigma^{S}S_{t}^{s,i}dW_{t}^{Q,1},\\ dX_{t}^{x,i,\varepsilon ,\sqrt{\gamma }h} & =\mu^{X}X_{t}^{x,i,\varepsilon ,\sqrt{\gamma }h}dt+\varepsilon \sigma^{X}X_{t}^{x,i,\varepsilon ,\sqrt{\gamma }h}(\rho \left[dW_{t}^{Q,2i-1}+mdt\right] \end{array}$$

(39) $$\begin{array}{cc} & \;+\sqrt{1-\rho^{2}}\left[dW_{t}^{Q,2i}+\sqrt{\gamma }h_{t}^{i}dt\right]), \end{array}$$

for i=1,…,d, where $W^{Q}=\left(W^{Q,1},\ldots ,W^{Q,2d}\right)$ defined by $dW_{t}^{Q,2i-1}=dW_{t}^{2i-1}-mdt$ and $dW_{t}^{Q,2i}=dW_{t}^{2i}-\sqrt{\gamma }h_{t}^{i}dt$, i=1,…,d is a Q-Brownian motion. We write $X^{x,\varepsilon ,h}=\left(X^{x,1,\varepsilon ,h},\ldots ,X^{x,d,\varepsilon ,h}\right)$.

We consider the case of a basket option of the nontradable assets, i.e., we set the payoff function $f:R^{d}\to R$ as $f\left(x\right)=\max\{\left(1/d\right)\sum_{i=1}^{d}x_{i}-K,0.0\}$. We assume that the riskfree rate r=0 for simplicity. Here, the buyer utility indifference price *p* is given by:

$$\begin{array}{cc} p & =-\frac{1}{\gamma (1-\rho^{2})}\log E\left[e^{-\gamma \left(1-\rho^{2}\right)f\left(X_{T}^{x,\varepsilon ,0}\right)}\right]\\ \; & =\underset{h\in A}{\inf}\left\{E\left[\frac{1}{2}\int_{0}^{T}{\left|h_{s}\right|}^{2}ds\right]+E\left[f\left(X_{T}^{x,\varepsilon ,\sqrt{\gamma }h}\right)\right]\right\}. \end{array}$$

We approximate the indifference price by the proposed method. Since $f\in C_{\mathrm{Lip}}\left(R^{d}\right)$, by Theorem 1, it holds that:

(40) $$\begin{array}{c} -\frac{1}{\gamma (1-\rho^{2})}\log E\left[\exp\left\{-\gamma \left(1-\rho^{2}\right)f\left(X_{T}^{x,\varepsilon ,0}\right)\right\}\right]\\ =E\left[f\left(X_{T}^{x,\varepsilon ,0}\right)\right]-\frac{1}{2}\gamma \left(1-\rho^{2}\right)\mathrm{Var}\left[f\left(X_{T}^{x,\varepsilon ,0}\right)\right]+O\left(\gamma^{2}{\left(1-\rho^{2}\right)}^{2}\varepsilon^{3}\right). \end{array}$$

In order to estimate the expansion error of (40), we compute the both sides by using the explicit solution of $X^{x,\varepsilon ,0}$ obtained by the Itô formula and Monte Carlo simulation with $M=10^{8}$ paths. Moreover, the approximation errors of the mean and variance terms are given by Corollary 1 as

(41) $$\begin{array}{cc} \; & E\left[f\left(X_{T}^{x,\varepsilon ,0}\right)\right]-\frac{1}{2}\gamma \left(1-\rho^{2}\right)\mathrm{Var}\left[f\left(X_{T}^{x,\varepsilon ,0}\right)\right]\\ = & E\left[f\left({\bar{X}}_{T}^{x,\varepsilon ,n}\right)W_{T}^{\varepsilon ,n}\right]-\frac{1}{2}\left\{E\left[f{\left({\bar{X}}_{T}^{x,\varepsilon ,n}\right)}^{2}W_{T}^{\varepsilon ,n}\right]-E{\left[f\left({\bar{X}}_{T}^{x,\varepsilon ,n}\right)W_{T}^{\varepsilon ,n}\right]}^{2}\right\}+O\left(\frac{\varepsilon^{3}}{n^{2}}\right), \end{array}$$

where ${\left\{{\bar{X}}_{t_{i}}^{x,\varepsilon ,n}\right\}}_{i=0,1,\ldots ,n}$ is the approximation process for $X^{x,\varepsilon }=X^{x,\varepsilon ,0}$ introduced by (27) and (28). To check the convergence rate of (41), we employ Monte Carlo simulation with $M=10^{8}$ paths to implement the proposed method, where the reference value (the left hand side of (41)) is obtained by the Itô formula and Monte Carlo simulation with $M=10^{8}$ paths.

#### 3.1.1. One-Dimensional Case

We perform the numerical experiment for the one-dimensional case. We set the parameters as d=1, T=1.0, x=100.0, K=100.0, γ=0.01, $\mu^{S}=\mu^{X}=0.0$, $\sigma^{S}=\sigma^{X}=0.2$, ρ=0.0.

First, we estimate the expansion error of (40) with respect to ε and γ. The mean-variance expansion (40) is referred to as “MV-expansion” in the following figures.

**Figure 1:**
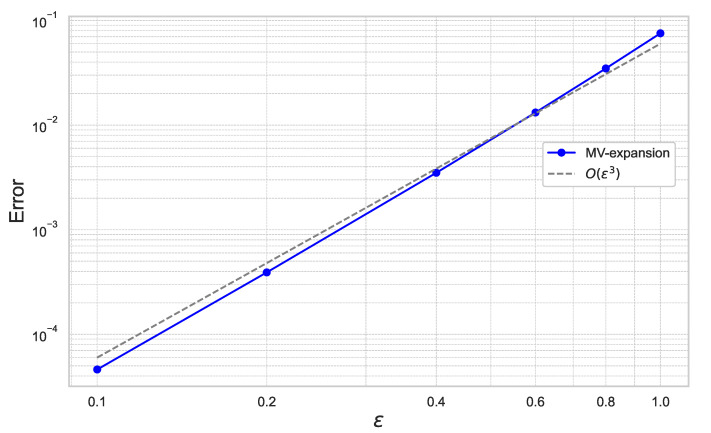
Expansion error of (40) for each ε with γ=0.01 under the one-dimensional Black–Scholes model.

Figure 1 plots the results for ε=0.1,0.2,0.4,0.6,0.8,1.0 for the fixed γ=0.01.

**Figure 2:**
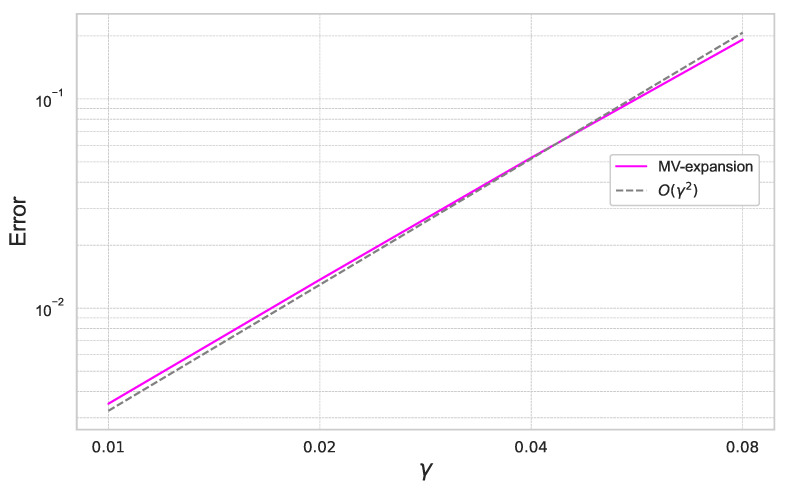
Expansion error of (40) for each γ with ε=0.4 under the one-dimensional Black–Scholes model.

Furthermore, we summarize the result for γ=0.01,0.02,0.04,0.08 for the fixed ε=0.4 in Figure 2.

In Figure 1 and Figure 2, we can check that the expansion error achieves the theoretical rate of convergence of $O(\gamma^{2}\varepsilon^{3})$.

**Figure 3:**
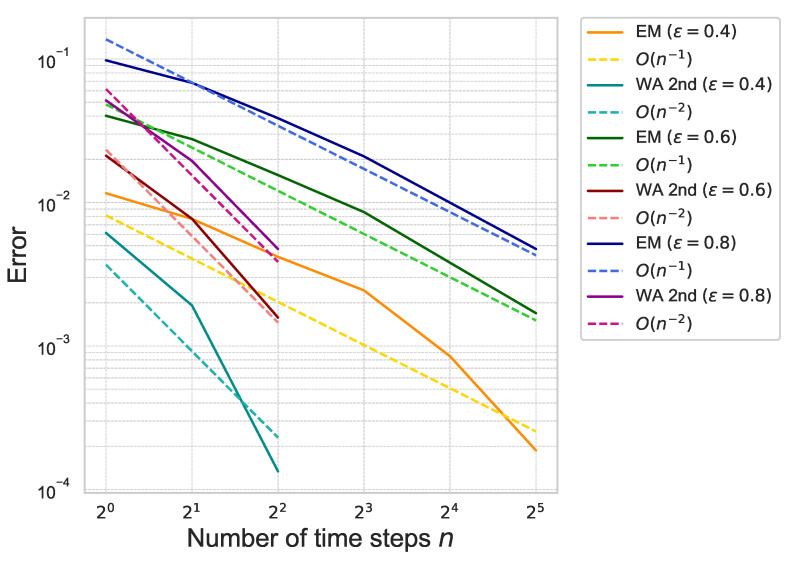
Weak approximation error of (41) for each ε with γ=0.01 under the one-dimensional Black–Scholes model.

Next, we estimate the weak approximation error of (41). The proposed second-order weak approximation method is referred to as “WA 2nd” in the following figures and tables. For comparison, we also compute the approximation error by using the Euler–Maruyama scheme, referred to as “EM”. The approximation error of (41) is plotted in Figure 3.

The figure shows that the error of “WA 2nd” decreases rapidly as the number of time steps increases compared to “EM”, which means that the proposed method achieves the second-order accuracy with respect to the number of time-steps *n*. The results are summarized in Table 1.

By the figures and the tables, we confirm that the proposed method achieves the theoretical rate of convergence with respect to γ, ε and *n*.

#### 3.1.2. 10-Dimensional Case

Now we consider a multidimensional case. We set the parameters as d=10, T=1.0, x=(100.0,…,100.0), K=100.0, γ=0.01, $\mu^{S}=\mu^{X}=0.0$, $\sigma^{S}=\sigma^{X}=0.2$, ρ=0.0.

**Figure 4:**
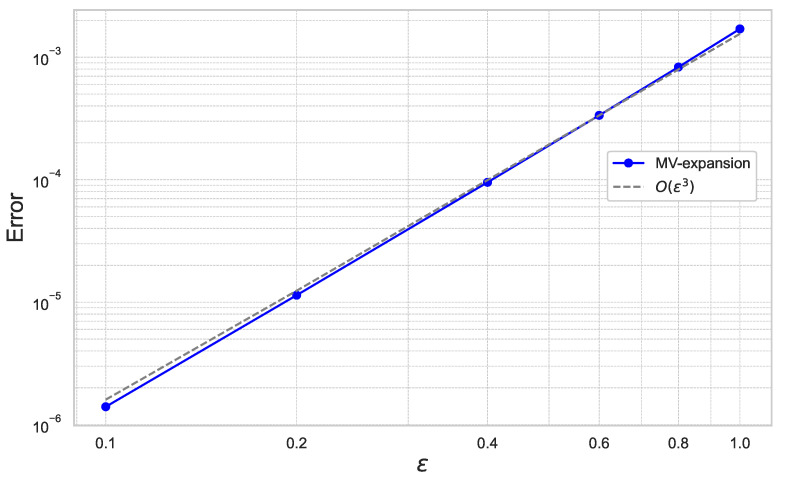
Expansion error of (40) for each ε with γ=0.01 under the 10-dimensional Black–Scholes model.

First, we estimate the expansion error of (40) with respect to ε and γ. Figure 4 plots the results for ε=0.1,0.2,0.4,0.6,0.8,1.0 for the fixed γ=0.01.

**Figure 5:**
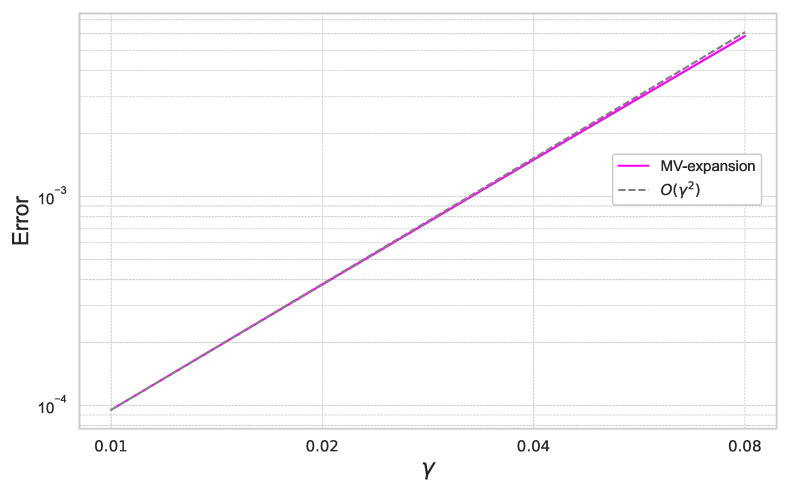
Expansion error of (40) for each γ with ε=0.4 under the 10-dimensional Black–Scholes model.

We perform the same experiment with the parameter γ=0.01,0.02,0.04,0.08 for the fixed ε=0.4. The result is summarized in Figure 5.

In Figure 4 and Figure 5, we can check that the expansion error achieves the theoretical rate of convergence of $O(\gamma^{2}\varepsilon^{3})$.

**Figure 6:**
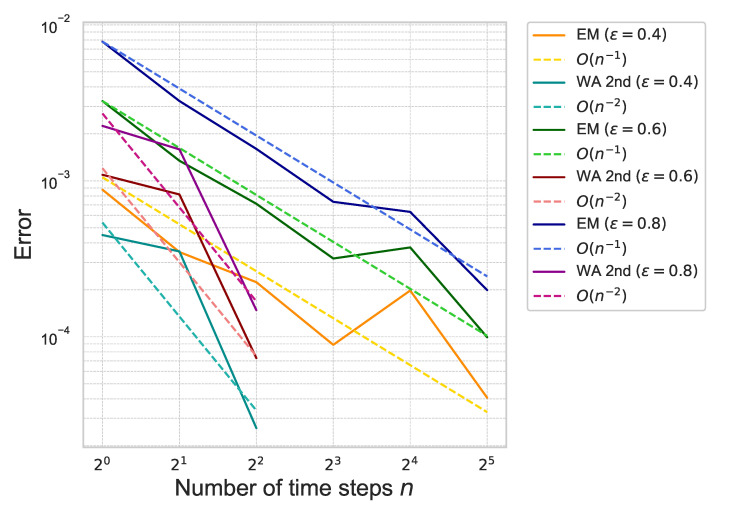
Weak approximation error of (41) for each ε with γ=0.01 under the 10-dimensional Black–Scholes model.

Next, we estimate the weak approximation error of (41). The discretization error is plotted in Figure 6.

The figure shows that the proposed method provides an accurate approximation for (41) with a small number of time steps compared to the Euler–Maruyama scheme. The results are summarized in Table 2.

By the figures and the table, we confirm that the proposed method achieves the theoretical rate of convergence.

#### 3.1.3. 100-Dimensional Case

Finally, we consider a higher-dimensional case. We set the parameters as d=10, T=1.0, x=(100.0,…,100.0), K=100.0, γ=0.01, $\mu^{S}=\mu^{X}=0.0$, $\sigma^{S}=\sigma^{X}=0.2$, ρ=0.0.

**Figure 7:**
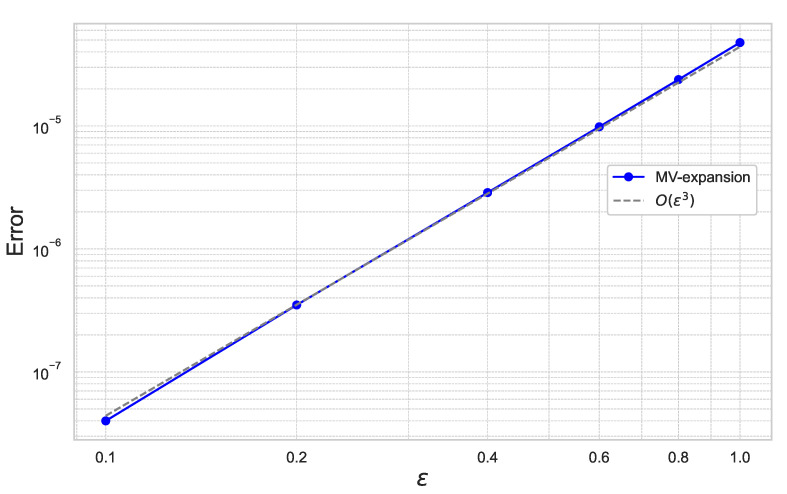
Expansion error of (40) for each ε with γ=0.01 under the 100-dimensional Black–Scholes model.

As the previous sections, we estimate the expansion error of (40). Figure 7 plots the results for ε=0.1,0.2,0.4,0.6,0.8,1.0 for the fixed γ=0.01.

**Figure 8:**
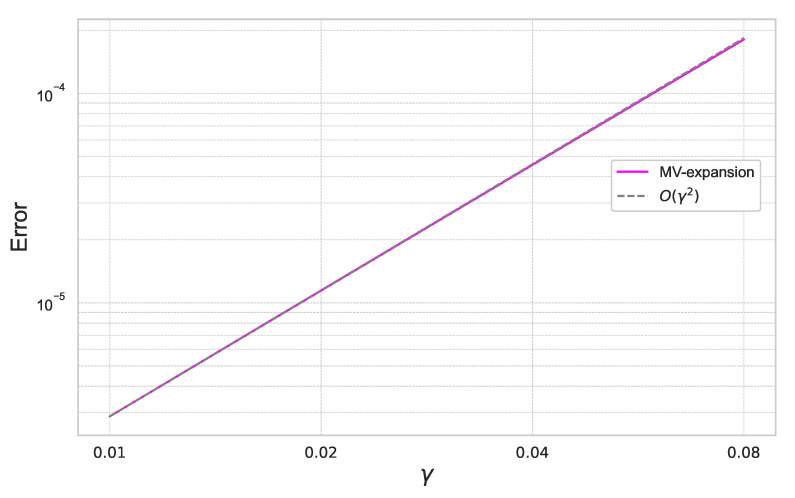
Expansion error of (40) for each γ with ε=0.4 under the 100-dimensional Black–Scholes model.

We also summarizes the result for each γ=0.01,0.02,0.04,0.08 for the fixed ε=0.4 in Figure 8.

In Figure 7 and Figure 8, we can check that the expansion error achieves the theoretical rate of convergence of $O(\gamma^{2}\varepsilon^{3})$.

Next, we estimate the weak approximation error of (41). We set the value we computed in the previous experiment as the reference value of the right hand side of (41). The discretization error is summarized in Table 3.

The table shows that the proposed method approximates (41) more accurately than the Euler–Maruyama scheme with a small number of time steps.

Throughout the numerical experiments, we confirm that the proposed method achieves the theoretical rate of convergence consistently with Theorem 1 and Corollary 1 in multidimensional settings with a Lipschitz continuous test function *f*.

### 3.2. Indifference Pricing under Constant Elasticity Model (CEV Model) with a Bounded Measurable Payoff Function

We consider the following two-dimensional SDE:

(42) $$\begin{array}{cc} dS_{t}^{s} & =\mu^{S}S_{t}^{s}dt+\sigma^{S}S_{t}^{s}dW_{t}^{1},\;\;S_{0}^{s}=s\in R, \end{array}$$

(43) $$\begin{array}{cc} dX_{t}^{x,\varepsilon } & =\mu^{X}X_{t}^{x,\varepsilon }dt+\varepsilon \sigma^{X}{\left(X_{t}^{x,\varepsilon }\right)}^{\beta }\left(\rho dW_{t}^{1}+\sqrt{1-\rho^{2}}dW_{t}^{2}\right),\;\;X_{0}^{x,\varepsilon }=x\in R, \end{array}$$

where $W=(W^{1},W^{2})$ is a P-dimensional Brownian motion. The dynamics of (43) is called the constant elasticity of variance model (CEV model), which is a generalized model of the Black–Scholes model.

We define Q∈M by:

(44) $$\begin{array}{c} \frac{dQ}{dP}=e^{mW_{T}^{1}-\frac{1}{2}m^{2}T+\int_{0}^{T}\sqrt{\gamma }h_{t}dW_{t}^{2}-\frac{1}{2}\int_{0}^{T}\gamma h_{t}^{2}dt}, \end{array}$$

for $m=\frac{r-\mu^{S}}{\sigma^{S}}$ and h∈A. Under a probability measure Q, we can rewrite the above SDE as:

(45) $$\begin{array}{cc} dS_{t}^{s} & =rS_{t}^{s}dt+\sigma^{S}S_{t}^{s}dW_{t}^{Q,1}, \end{array}$$

(46) $$\begin{array}{cc} dX_{t}^{x,\varepsilon ,\sqrt{\gamma }h} & =\mu^{X}X_{t}^{x,\varepsilon ,\sqrt{\gamma }h}dt+\varepsilon \sigma^{X}{\left(X_{t}^{x,\varepsilon ,\sqrt{\gamma }h}\right)}^{\beta }\left(\rho \left[dW_{t}^{Q,1}+mdt\right]+\sqrt{1-\rho^{2}}\left[dW_{t}^{Q,2}+\sqrt{\gamma }h_{t}dt\right]\right), \end{array}$$

where $W^{Q}=\left(W^{Q,1},W^{Q,2}\right)$ defined by $dW_{t}^{Q,1}=dW_{t}^{1}-mdt$ and $dW_{t}^{Q,2}=dW_{t}^{2}-\sqrt{\gamma }h_{t}dt$ is a Q-Brownian motion.

As the previous section, we assume that the riskfree rate r=0 for simplicity. We consider the case of a digital option of the nontradable asset, i.e., we set the payoff function f:R→R as $f\left(x\right)=1_{\left\{x>K\right\}}$.

Here, the buyer utility indifference price *p* is given by:

(47) $$\begin{array}{cc} p & =-\frac{1}{\gamma (1-\rho^{2})}\log E\left[e^{-\gamma \left(1-\rho^{2}\right)f\left(X_{T}^{x,\varepsilon ,0}\right)}\right]=\underset{h\in A}{\inf}\left\{E\left[\frac{1}{2}\int_{0}^{T}{\left|h_{s}\right|}^{2}ds\right]+E\left[f\left(X_{T}^{x,\varepsilon ,\sqrt{\gamma }h}\right)\right]\right\}. \end{array}$$

We approximate the indifference price by the proposed method. Since $f\in B_{b}\left(R\right)$, it holds that:

(48) $$\begin{array}{cc} \; & -\frac{1}{\gamma (1-\rho^{2})}\log E\left[\exp\left\{-\gamma \left(1-\rho^{2}\right)f\left(X_{T}^{x,\varepsilon ,0}\right)\right\}\right] \end{array}$$

(49) $$\begin{array}{c} =E\left[f\left(X_{T}^{x,\varepsilon ,0}\right)\right]-\frac{1}{2}\gamma \left(1-\rho^{2}\right)\mathrm{Var}\left[f\left(X_{T}^{x,\varepsilon ,0}\right)\right]+O\left(\gamma^{2}{\left(1-\rho^{2}\right)}^{2}\right). \end{array}$$

To estimate the expansion error of (49), we compute both sides by using the Euler–Maruyama discretization scheme with $n=2^{10}$ time steps and Monte Carlo simulation with $M=2\times 10^{9}$ paths. The mean-variance expansion (49) is referred to as “MV-expansion (EM $n=2^{10}$)” in the following figures.

Moreover, the approximation errors of the mean and variance terms are given by Corollary 1 as:

(50) $$\begin{array}{c} E\left[f\left(X_{T}^{x,\varepsilon ,0}\right)\right]-\frac{1}{2}\gamma \left(1-\rho^{2}\right)\mathrm{Var}\left[f\left(X_{T}^{x,\varepsilon ,0}\right)\right]\\ =E\left[f\left({\bar{X}}_{T}^{x,\varepsilon ,n}\right)W_{T}^{\varepsilon ,n}\right]-\frac{1}{2}\left\{E\left[f{\left({\bar{X}}_{T}^{x,\varepsilon ,n}\right)}^{2}W_{T}^{\varepsilon ,n}\right]-E{\left[f\left({\bar{X}}_{T}^{x,\varepsilon ,n}\right)W_{T}^{\varepsilon ,n}\right]}^{2}\right\}+O\left(\frac{\varepsilon^{2}}{n^{2}}\right), \end{array}$$

where ${\left\{{\bar{X}}_{t_{i}}^{x,\varepsilon ,n}\right\}}_{i=0,1,\ldots ,n}$ is the approximation process for $X^{x,\varepsilon ,0}$ introduced by (27) and (28).

To check the convergence rate of (50), we employ Monte Carlo simulation with $M=2\times 10^{9}$ paths to implement the proposed method, where the reference value (the left hand side of (50)) is obtained by using the Euler–Maruyama discretization scheme with $n=2^{10}$ time steps and Monte Carlo simulation with $M=2\times 10^{9}$ paths.

**Figure 9:**
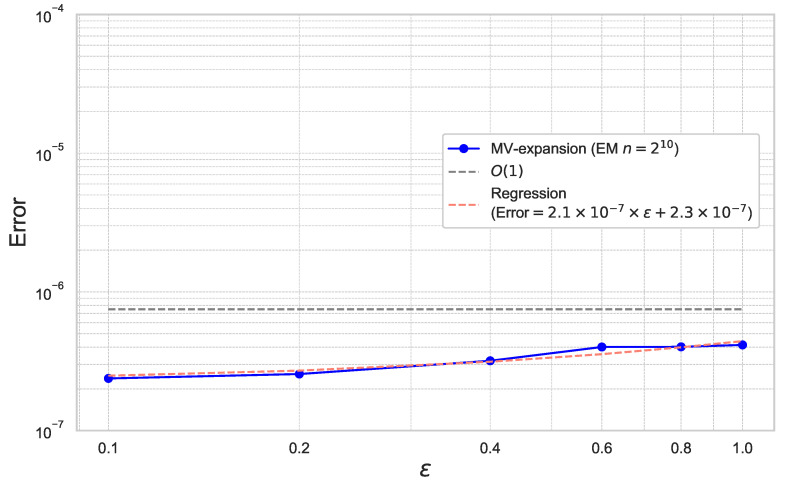
Expansion error of (49) for each ε with γ=0.025 under the CEV model.

We set the parameters as T=2.0, x=100.0, K=100.0, $\mu^{S}=\mu^{X}=0.0$, $\sigma^{S}=\sigma^{X}=0.3$, ρ=0.0, β=0.5. We estimate the approximation error of (49) with respect to γ. Figure 9 plots the results for ε=0.1,0.2,0.4,0.6,0.8,1.0 for the fixed γ=0.025.

In Figure 9, a linear regression line (which is referred to as “Regression” in Figure 9) is added to confirm the empirical convergence rate. We can check that the coefficient of ε is quite small and the regression line seems to be flat. By the experiment, we confirm that the expansion error is consistent with Theorem 1.

Next, we perform the same experiments with the parameter γ=0.0125,0.025,0.05,0.1 for the fixed ε=0.4.

**Figure 10:**
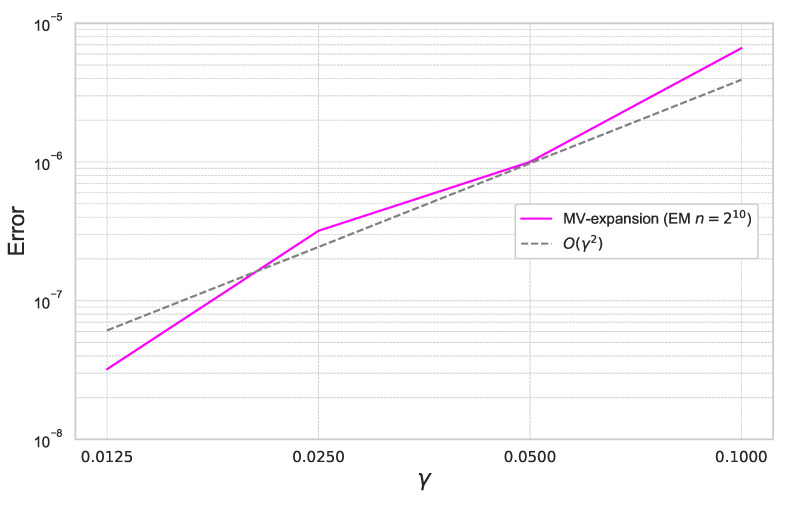
Expansion error of (49) for each γ with ε=0.4 under the CEV model.

In Figure 10, we can check that the expansion error achieves the theoretical rate of convergence of $O(\gamma^{2})$.

Finally, we estimate the weak approximation error of (50). The result is summarized in the next table.

Table 4 shows that the proposed method provides an accurate approximation compared to the Euler–Maruyama scheme.

### 3.3. Indifference Pricing under Stochastic Volatility Model with a Lipschitz Payoff Function

We consider the following 3d-dimensional SDE:

(51) $$\begin{array}{cc} dS_{t}^{s,i} & =\mu^{S}S_{t}^{s,i}dt+\sigma^{S}S_{t}^{s,i}dW_{t}^{3i-2},\;\;S_{0}^{s,i}=s_{i}\in R, \end{array}$$

(52) $$\begin{array}{cc} dX_{t}^{x,i,\varepsilon } & =\mu^{X}X_{t}^{x,i,\varepsilon }dt+\varepsilon V_{t}^{v,i,\varepsilon }X_{t}^{x,i,\varepsilon }\left(\rho dW_{t}^{3i-2}+\sqrt{1-\rho^{2}}dW_{t}^{3i-1}\right),\;\;X_{0}^{x,i,\varepsilon }=x_{i}\in R, \end{array}$$

(53) $$\begin{array}{cc} dV_{t}^{v,i,\varepsilon } & =\varepsilon \nu V_{t}^{v,i,\varepsilon }\left(\tilde{\rho }dW_{t}^{3i-1}+\sqrt{1-{\tilde{\rho }}^{2}}dW_{t}^{3i}\right),\;\;V_{0}^{v,i,\varepsilon }=v\in R, \end{array}$$

for i=1,…,d where $W=(W^{1},\ldots ,W^{3d})$ is a P-dimensional Brownian motion. The above SDE represents the stochastic volatility model and is widely used by practitioners in financial institutions.

We define Q∈M by:

(54) $$\begin{array}{c} \frac{dQ}{dP}=e^{\sum_{i=1}^{d}\left(mW_{T}^{3i-2}-\frac{1}{2}m^{2}T\right)+\sum_{i=1}^{d}\int_{0}^{T}\sqrt{\gamma }h_{t}^{i}dW_{t}^{3i-1}-\frac{1}{2}\int_{0}^{T}\gamma |h_{t}{|}^{2}dt+\sum_{i=1}^{d}\int_{0}^{T}\sqrt{\gamma }k_{t}^{i}dW_{t}^{3i}-\frac{1}{2}\int_{0}^{T}\gamma {\left|k_{t}\right|}^{2}dt}, \end{array}$$

for $m=\frac{r-\mu^{S}}{\sigma^{S}}$ and h,k∈A. Under a probability measure Q, we can rewrite the above SDE as:

(55) $$\begin{array}{cc} dS_{t}^{s,i} & =rS_{t}^{s,i}dt+\sigma^{S}S_{t}^{s,i}dW_{t}^{Q,3i-2},\\ dX_{t}^{x,i,\varepsilon ,\sqrt{\gamma }h,\sqrt{\gamma }k} & =\mu^{X}X_{t}^{x,i,\varepsilon ,\sqrt{\gamma }h,\sqrt{\gamma }k}dt+\varepsilon V_{t}^{v,i,\varepsilon ,\sqrt{\gamma }h,\sqrt{\gamma }k}X_{t}^{x,i,\varepsilon ,\sqrt{\gamma }h,\sqrt{\gamma }k} \end{array}$$

(56) $$\begin{array}{cc} & \;\times (\rho \left[dW_{t}^{Q,3i-2}+mdt\right]+\sqrt{1-\rho^{2}}\left[dW_{t}^{Q,3i-1}+\sqrt{\gamma }h_{t}^{i}dt\right), \end{array}$$

(57) $$\begin{array}{cc} dV_{t}^{v,i,\varepsilon ,\sqrt{\gamma }h,\sqrt{\gamma }k} & =\varepsilon \nu V_{t}^{v,i,\varepsilon ,\sqrt{\gamma }h,\sqrt{\gamma }k}\left(\tilde{\rho }\left[dW_{t}^{Q,3i-1}+\sqrt{\gamma }h_{t}^{i}dt\right]+\sqrt{1-{\tilde{\rho }}^{2}}\left[dW_{t}^{Q,3i}+\sqrt{\gamma }k_{t}^{i}dt\right]\right), \end{array}$$

for i=1,…,d, where $W^{Q}=\left(W^{Q,1},\ldots ,W^{Q,3d}\right)$ defined by $dW_{t}^{Q,3i-2}=dW_{t}^{3i-2}-mdt$, $dW_{t}^{Q,3i-1}=dW_{t}^{3i-1}-\sqrt{\gamma }h_{t}^{i}dt$ and $dW_{t}^{Q,3i}=dW_{t}^{3i}-\sqrt{\gamma }k_{t}^{i}dt$, i=1,…,d is a Q-Brownian motion. We write $X^{x,\varepsilon ,h}=\left(X^{x,1,\varepsilon ,h},\ldots ,X^{x,d,\varepsilon ,h}\right)$ and $V^{v,\varepsilon ,h}=\left(V^{v,1,\varepsilon ,h},\ldots ,V^{v,d,\varepsilon ,h}\right)$.

We consider the case of a maximum option of the nontradable assets, i.e., we set the payoff function $f:R^{d}\to R$ as $f\left(x\right)=\max\{\max\left\{x_{1}-K,0.0\right\},\ldots ,\max\left\{x_{d}-K,0.0\right\}\}$. We assume that the riskfree rate r=0 as the previous examples.

Here, the buyer utility indifference price is given by:

(58) $$\begin{array}{cc} p & =-\frac{1}{\gamma (1-\rho^{2})}\log E\left[e^{-\gamma \left(1-\rho^{2}\right)f\left(X_{T}^{x,\varepsilon ,0,0}\right)}\right]\\ & =\underset{h,k\in A}{\inf}\left\{E\left[\frac{1}{2}\int_{0}^{T}\left(\right|h_{s}{|}^{2}+|k_{s}{|}^{2})ds\right]+E\left[f\left(X_{T}^{x,\varepsilon ,\sqrt{\gamma }h,\sqrt{\gamma }k}\right)\right]\right\}. \end{array}$$

Since $f\in C_{\mathrm{Lip}}\left(R^{d}\right)$, by Theorem 1, we have:

(59) $$\begin{array}{cc} \; & -\frac{1}{\gamma (1-\rho^{2})}\log E\left[e^{-\gamma \left(1-\rho^{2}\right)f\left(X_{T}^{x,\varepsilon ,0,0}\right)}\right]\\ \; & =E\left[f\left(X_{T}^{x,\varepsilon ,0,0}\right)\right]-\frac{1}{2}\gamma \left(1-\rho^{2}\right)\mathrm{Var}\left[f\left(X_{T}^{x,\varepsilon ,0,0}\right)\right]+O\left(\gamma^{2}\left(1-\rho^{2}\right)\varepsilon^{3}\right). \end{array}$$

We check the expansion error of (59) by computing the both sides by using the Euler–Maruyama discretization scheme with $n=2^{10}$ time steps and Monte Carlo simulation with $M=10^{8}$ paths. The mean-variance expansion (59) is referred to as “MV-expansion (EM $n=2^{10}$)” in the following figures.

Moreover, the approximation errors of the mean and variance terms are given by Corollary 1 as:

(60) $$\begin{array}{c} E\left[f\left(X_{T}^{x,\varepsilon ,0,0}\right)\right]-\frac{1}{2}\gamma \left(1-\rho^{2}\right)\mathrm{Var}\left[f\left(X_{T}^{x,\varepsilon ,0,0}\right)\right]\\ =E\left[f\left({\bar{X}}_{T}^{x,\varepsilon ,n}\right)W_{T}^{\varepsilon ,n}\right]-\frac{1}{2}\left\{E\left[f{\left({\bar{X}}_{T}^{x,\varepsilon ,n}\right)}^{2}W_{T}^{\varepsilon ,n}\right]-E{\left[f\left({\bar{X}}_{T}^{x,\varepsilon ,n}\right)W_{T}^{\varepsilon ,n}\right]}^{2}\right\}+O\left(\frac{\varepsilon^{2}}{n^{2}}\right), \end{array}$$

where ${\left\{{\bar{X}}_{t_{i}}^{x,\varepsilon ,n}\right\}}_{i=0,1,\ldots ,n}$ is the approximation process for $X^{x,\varepsilon ,0,0}$ introduced by (27) and (28).

To check the convergence rate of (60), we implement the proposed method by Monte Carlo simulation with $M=10^{8}$ paths, where the reference value (the left hand side of (60)) is obtained by using the Euler–Maruyama discretization scheme with $n=2^{10}$ time steps and Monte Carlo simulation with $M=10^{8}$ paths.

We set the parameters as d=10, T=1.0, x=(100.0,…,100.0), v=(0.2,…,0.2), K=100.0, γ=0.01, $\mu^{S}=\mu^{X}=0.0$, $\sigma^{S}=0.2$, ν=0.1, ρ=0.0, $\tilde{\rho }=-0.5$. We estimate the expansion error of (59) with respect to ε and γ.

**Figure 11:**
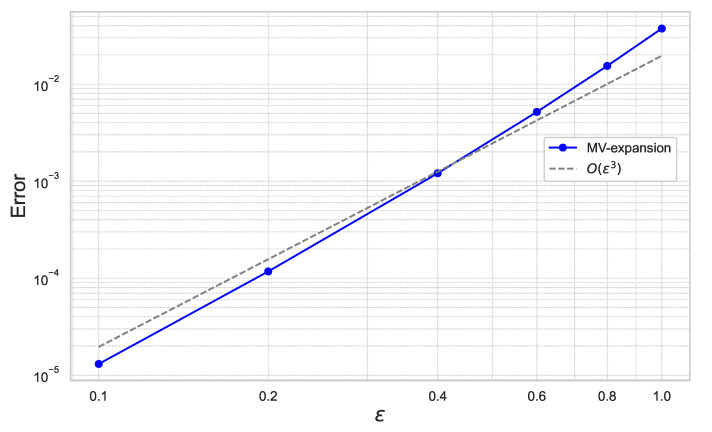
Expansion error of (59) for each ε with γ=0.01 under the 20-dimensional stochastic volatility model.

Figure 11 plots the results for ε=0.1,0.2,0.4,0.6,0.8,1.0 for the fixed γ=0.01.

**Figure 12:**
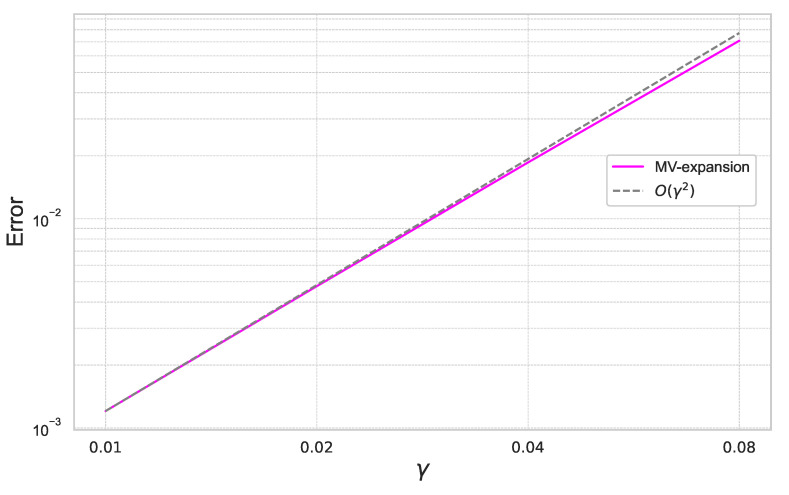
Expansion error of (59) for each γ with ε=0.4 under the 20-dimensional stochastic volatility model.

Furthermore, we summarize the result for γ=0.01,0.02,0.04,0.08 for the fixed ε=0.4 in Figure 12.

In Figure 11 and Figure 12, we can check that the expansion error achieves the theoretical rate of convergence of $O(\gamma^{2}\varepsilon^{3})$.

**Figure 13:**
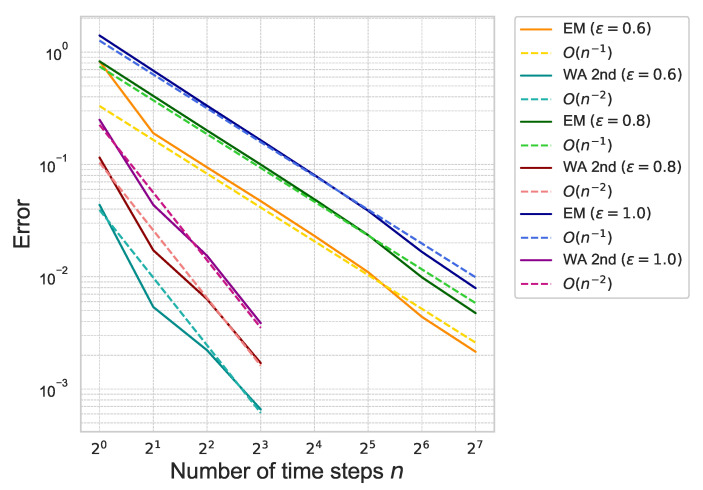
Weak approximation error of (60) for each ε with γ=0.01 under the 20-dimensional stochastic volatility model.

Next, we estimate the weak approximation error of (60). The approximation error of (60) is plotted in Figure 13.

The figure shows that the error of “WA 2nd” decreases rapidly as the number of time steps increases compared to “EM”, which means that the proposed method achieves the second-order accuracy with respect to the number of time-steps *n*. The results are summarized in Table 5.

Throughout the numerical experiments, we confirm that the proposed method provides a consistent result with Theorem 1 and Corollary 1 even when *f* is a non-smooth function.

## 4. Conclusions

In the paper, we showed a precise error estimate of the mean-variance expansion of the stochastic control problem under general conditions on the terminal test function based on asymptotic expansion and Malliavin calculus. In particular, we proved that the expansion error depends on the smoothness of the test function, which is an extension of [11,14,15]. Moreover, an efficient algorithm has been introduced based on the asymptotic expansion method and weak approximation for the small noise diffusion. Numerical experiments confirmed the theoretical convergence rate of γ, the small noise parameter ε and the number of time-steps. For future work, it is worth considering to apply the proposed method to important problems in statistical physics, such as the rare event simulation studied by [7,8].

## Figures and Tables

**Table 1:** Numerical error of (41) for each ε with γ=0.01 under the one-dimensional Black–Scholes model.

	ε=0.4	ε=0.6	ε=0.8
EM ($n=2^{5}$)	$1.9\times 10^{-4}$	$1.7\times 10^{-3}$	$4.7\times 10^{-3}$
WA 2nd ($n=2^{2}$)	$1.3\times 10^{-4}$	$1.6\times 10^{-3}$	$4.7\times 10^{-3}$

**Table 2:** Numerical error of (41) for each ε with γ=0.01 under the 10-dimensional Black–Scholes model.

	ε=0.4	ε=0.6	ε=0.8
EM ($n=2^{5}$)	$4.1\times 10^{-5}$	$9.9\times 10^{-5}$	$2.0\times 10^{-4}$
WA 2nd ($n=2^{2}$)	$2.6\times 10^{-5}$	$7.3\times 10^{-5}$	$1.5\times 10^{-4}$

**Table 3:** Numerical error of (41) for each ε with γ=0.01 under the 100-dimensional Black–Scholes model.

	ε=0.4	ε=0.6	ε=0.8
EM ($n=2^{4}$)	$8.1\times 10^{-5}$	$1.8\times 10^{-5}$	$3.5\times 10^{-4}$
WA 2nd ($n=2^{1}$)	$9.8\times 10^{-6}$	$4.0\times 10^{-7}$	$2.6\times 10^{-5}$

**Table 4:** Numerical error of (50) for each ε with γ=0.025 under the CEV model.

	ε=0.4	ε=0.6	ε=0.8
EM ($n=2^{4}$)	$8.5\times 10^{-5}$	$1.3\times 10^{-4}$	$1.9\times 10^{-4}$
WA 2nd ($n=2^{0}$)	$4.7\times 10^{-5}$	$4.6\times 10^{-5}$	$4.5\times 10^{-5}$

**Table 5:** Numerical error of (60) for each ε with γ=0.01 under the 20-dimensional stochastic volatility model.

	ε=0.6	ε=0.8	ε=1.0
EM ($n=2^{7}$)	$4.7\times 10^{-3}$	$7.9\times 10^{-3}$	$1.1\times 10^{-2}$
WA 2nd ($n=2^{3}$)	$6.6\times 10^{-4}$	$1.7\times 10^{-3}$	$3.9\times 10^{-3}$

## Data Availability

No new data were created or analyzed in this study. Data sharing is not applicable to this article.

## Abbreviations

The following abbreviations are used in this manuscript:

SDE	Stochastic Differential Equation
